# Influence of the leukoreduction moment of blood components on the clinical outcomes of transfused patients in the emergency department

**DOI:** 10.1590/0034-7167-2023-0293

**Published:** 2024-08-26

**Authors:** Natasha Dejigov Monteiro da Silva, Ana Catharina Herbst, Milena Raquel André, Lilia de Souza Nogueira

**Affiliations:** I Universidade de São Paulo. São Paulo, São Paulo, Brazil

**Keywords:** Blood Transfusion, Leukocyte Reduction Procedures, Transfusion Reaction, Clinical Evolution, Emergency Medical Service, Transfusión Sanguínea, Procedimientos de Reducción del Leucocitos, Reacción a la Transfusión, Evolución Clínica, Servicios Médicos de Urgencia

## Abstract

**Objectives:**

to investigate the influence of the leukoreduction moment (preor post-storage) of blood components on the clinical outcomes of patients transfused in the emergency department.

**Methods:**

retrospective cohort study of patients aged 18 years or older who received preor post-storage leukoreduced red blood cell or platelet concentrate in the emergency department and remained in the institution for more than 24 hours. A generalized mixed-effects model was applied in the analyses.

**Results:**

in a sample of 373 patients (63.27% male, mean age 54.83) and 643 transfusions (69.98% red blood cell), it was identified that the leukoreduction moment influenced the length of hospital stay (p<0.009), but was not dependent on the transfused blood component (p=0.124). The leukoreduction moment had no effect (p>0.050) on transfusion reactions, healthcare-associated infections, or mortality.

**Conclusions:**

patients who received pre-storage leukoreduced blood components in the emergency department had a shorter length of hospital stay.

## RESUMEN

**Objetivos:**

verificar la influencia del momento de la leucorreducción (pre o post-almacenamiento) de hemocomponentes en la evolución clínica de pacientes transfundidos en la emergencia.

**Métodos:**

cohorte retrospectiva de pacientes de 18 años o más que recibieron, en el departamento de emergencia, concentrado de eritrocitos o plaquetas leucorreducidas pre o post-almacenamiento. Se aplicó un modelo de efectos mixtos generalizado en los análisis.

**Resultados:**

en la muestra de 373 pacientes (63,27% hombres, edad media 54,83) y 643 transfusiones (69,98% concentrado de eritrocitos) se identificó que el momento de la leucorreducción influyó en el tiempo de internación hospitalaria de los pacientes (p<0,009), pero no dependió del hemocomponente transfundido (p=0,124). El momento de la leucorreducción no tuvo efecto (p>0,050) en las variables de reacción transfusional, infección relacionada con la atención de la salud y óbito.

**Conclusiones:**

los pacientes que recibieron hemocomponentes leucorreducidos pre-almacenamiento en la emergencia presentaron un menor tiempo de internación hospitalaria.

## INTRODUCTION

Blood component transfusion is a crucial therapy for treating patients, especially critically ill ones, with the goal of achieving clinical improvement, such as increased tissue oxygenation, prevention of hemorrhage, or cessation of bleeding^(1-3)^. Blood components are products generated from whole blood and classified into red blood cells (RBCs), platelet concentrates (PCs), fresh frozen plasma (FFP), and cryoprecipitate (Cryo)^(2,4)^.

During the collection, processing, and storage stages of blood components, the release of inflammatory mediators resulting from leukocyte degradation occurs. During transfusion, leukocyte antigens and metabolically active cells capable of proliferating and producing immunological modifiers are introduced, which affect the recipient. The recipient will respond to the transfusion by producing their own immunological mediators, leading to the occurrence of transfusion reactions (TRs)^(4-7)^. According to the *Agence Régionale de Santé Île-de-France*, the estimated rate of TRs is 3 to 5 per 1,000 transfusions^(8)^, with RBCs and PCs being responsible for most of them^(3,8-9)^.

To reduce adverse transfusion effects, especially febrile non-hemolytic reactions (FNHR), alloimmunization to the Human Leukocyte Antigen (HLA) system, and transfusion-related acute lung injury (TRALI), one of the adopted procedures is leukoreduction of RBCs and PCs, which can be performed using filters at two moments: pre-storage (bench or inline) or post-storage (bedside)^(10-12)^. Bench and inline filtrations are performed in the Hemotherapy Service within 48 hours and between 2 to 24 hours after collection, respectively. Bedside filtration is performed at the time of the transfusion^(12)^. FFP and Cryo do not require leukoreduction, as there are not enough leukocytes in these components to be harmful to the recipient.

In the emergency department, the use of blood components is frequent and justified by the need to assist in volume replacement, control bleeding, and restore tissue oxygenation. There is evidence of the benefits of pre-storage leukoreduction compared to post-storage in terms of reducing the occurrence of TRs and infections in patients transfused in different hospital sectors^(13-19)^. However, analyses of patients who received leukoreduced blood components in the emergency department are scarce in the literature.

It is also important to emphasize that the role of the nurse and the entire healthcare team during blood component transfusion is crucial to ensuring the safety and efficacy of the procedure, especially for patients in critical health situations. In this context, implementing processes that reduce the occurrence of adverse transfusion effects not only benefits the patient but also the healthcare facility by reducing the workload of the healthcare team, lowering the consumption of resources for treating complications, and potentially shortening hospital stays^(20)^.

Considering the importance of leukoreduction in reducing adverse transfusion effects and identifying knowledge gaps regarding the possible impact of the leukoreduction moment (preor post-storage) on the clinical outcomes of patients who received blood components during emergency care, the relevance of the present research is justified.

## OBJECTIVES

To investigate the influence of the leukoreduction moment of blood components on the clinical outcomes of patients transfused in the emergency department.

## METHODS

### Ethical Aspects

This research was approved by the Ethics Committees of the participating institutions. Since it involved retrospective data collection with documentary analysis, the requirement for obtaining informed consent was waived, with the assurance that researchers would maintain the confidentiality and reliability of the collected information.

### Design, Period, and Study Location

This is a retrospective cohort study guided by the Strengthening the Reporting of Observational Studies in Epidemiology (STROBE) tool. Data collection was carried out in three institutes of the *Hospital das Clínicas da Faculdade de Medicina da Universidade de São Paulo* (HCFMUSP): *Instituto do Câncer do Estado de São Paulo* (ICESP), specializing in oncology; *Instituto do Coração* (InCor), specializing in cardiopulmonology; and *Instituto Central* (ICHC), which serves various specialties and is a reference center for trauma care.

### Population or Sample; Inclusion and Exclusion Criteria

The convenience sample included patients aged 18 years or older who received, in the emergency department of one of the institutes, transfusions of allogeneic leukoreduced RBCs and/or PCs, either preor post-storage, between January 1, 2017, and December 31, 2020, and who remained hospitalized for at least 24 hours. Patients with a do-not-resuscitate order, brain death, and/or a diagnosis of sepsis at hospital admission were excluded from the sample.

### Study Protocol

The independent variable in this study was the leukoreduction moment, categorized as pre-storage (inline or bench) or post-storage (bedside). The dependent variables related to the clinical outcomes of patients included the occurrence of TRs (categorized as yes or no based on the identification of FNHR, transfusion-related hypotension, TRALI, alloimmunization/HLA, or other transfusion-related symptoms); the presence of healthcare-associated infections (HAIs) within 72 hours post-transfusion (categorized as yes or no); length of hospital stay (LOS) (measured in days); and in-hospital mortality (categorized as yes or no).

For data collection, the Blood Bank of the *Fundação Pró-Sangue do Hemocentro de São Paulo* (FPS-HSP) was requested to provide a database of blood component requisitions and dispensations during the study period. This database was used to extract information regarding blood components and previous transfusions and reactions of the patients. This information was cross-referenced with the electronic medical records of patients admitted to the participating institutes, applying the study’s eligibility criteria, and collecting the relevant research variables.

It is important to note that pre-storage filtration (inline or bench) was performed at FPS-HSP. For inline filtration, the Trima Accel^®^ automated blood collection system was used, with a filtration capacity of more than 5 x 10^6 leukocytes per unit. For bench filtration, the BIOR 01 PLUS BBS PF filter (for RBCs) and the BIOP 10 Plus BBSS PF filter (for PCs), both from Fresenius^®^, were used, with a residual leukocyte level of less than 2 x 10^5 per unit. Bedside filtration was performed at the Institutes at the time of transfusion using the RC1VAE (Auto Prime) filter from Haemonetics^®^ (for RBCs), with a residual capacity of less than 2 x 10^5 leukocytes per unit, and the PL3VAE filter from Haemonetics^®^ (for PCs), with platelet recovery of over 90% and a residual leukocyte level of less than 2 x 10^5 per transfusion.

Information on the occurrence of TRs was collected from the Notification System for Health Surveillance (NOTIVISA) and the Health Surveillance Center (CVS-4), which contain mandatory records of the final destination of blood components prepared for transfusion, as well as the patients’ electronic medical records. Information on HAIs was obtained by searching for culture test records performed within 72 hours post-transfusion, and the validation of infection criteria was carried out in conjunction with the Hospital Infection Control Committees of the Institutes. The LOS and the discharge condition were identified from the patients’ electronic medical records.

Considering the complexity of retrieving information, which involved consulting different sources, the study period (2017 to 2020), and access restrictions to the Institutes due to the COVID-19 pandemic, data collection lasted two years (2021 to 2022).

### Analysis of Results and Statistics

The collected data were entered into Microsoft Excel^®^ 2019 and analyzed by a statistical professional using R version 4.3.1. Considering that each patient could have received more than one transfusion during their stay in the emergency department, a generalized mixed-effects model for the binomial family was applied to verify the influence of the leukoreduction moment on each dependent variable of the study, controlled for the transfused blood component (RBCs or PCs). The significance level adopted in all analyses was 5%.

## RESULTS

During the study period, 373 patients received blood components in the emergency department, with the majority being male (63.27%) and white (68.84%). The mean and median ages were 54.83 and 58 years, respectively. Regarding the diagnosis at emergency admission, neoplasms (n=118; 31.63%) and diseases of the blood and hematopoietic organs (n=112; 30.03%) were most prevalent. The most common blood type was O Rh positive or negative (46.92%), followed by A Rh positive or negative (37.26%). A total of 303 (81.23%) patients had previously received a transfusion, and of these, 269 (88.78%) reported having experienced a transfusion reaction.

Out of the 643 transfusions performed in the emergency department, RBCs were the most administered blood component (69.98%), and bedside filtration was the most utilized method (86.47%). Among the 144 transfusion justifications described, anemia (61.80%) and active bleeding (27.08%) were the most prevalent. The average number of transfusions per patient was 1.45 (SD 0.93).

In the sample, 52 (8.09%) TRs were identified, with 41 described as undefined transfusion reactions, 8 allergic reactions, 2 FNHR, and 1 transfusion-associated dyspnea. A total of 6 patients presented with HAIs within 72 hours of transfusion, predominantly urinary tract infections. The average of LOS was 4.98 (SD 8.18) days, ranging from 0.57 to 88.47 days. Regarding hospital outcomes, 35 (9.38%) patients did not survive.

**Figure 1:**
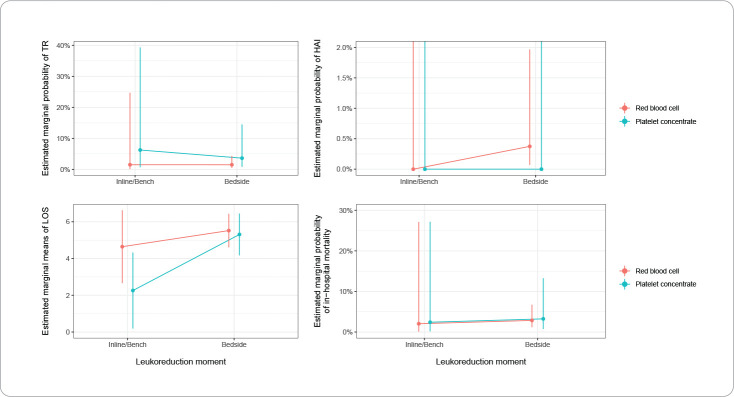
Estimates of transfusion reaction, healthcare-associated infection, length of hospital stay and in-hospital mortality according to the leukoreduction moment and type of blood component, São Paulo, Brazil, 2017-2020 TR – Transfusion Reaction; HAI – Healthcare-Associated Infection; LOS – Length of hospital stay.

In the application of the generalized mixed-effects model, the cluster of 373 patients was considered for the 643 transfusion observations. The data in Tables 1 and Figure 1 show no significant evidence that the leukoreduction moment affected the occurrence of RTs (p=0.786), regardless of the transfused blood component (p=0.796).

**Table 1:** Influence of the leukoreduction moment on the occurrence of transfusion reaction, São Paulo, Brazil, 2017-2020

	X^2^	df	*p* value^*^
(Intercept)	41.152	1	<0.001
Leukoreduction moment	0.074	1	0.786
Blood component	1.263	1	0.261
Leukoreduction moment:blood component	0.067	1	0.796

X^2^ - Chi-Square; df - Degrees of Freedom;

* Generalized mixed-effects model for binomial family.

*TR - Transfusion Reaction; HAI - Healthcare-Associated Infection; LOS - Length of hospital stay.*

For the analysis of the presence of HAIs within 72 hours post-transfusion, the findings showed that the leukoreduction moment had a similar effect on this outcome (p=1.000), regardless of the administered blood component (p=1.000) (Table 2, Figure 1).

**Table 2:** Influence of the leukoreduction moment on the presence of healthcare-associated infection, São Paulo, Brazil, 2017-2020

	X^2^	df	*p* value^*^
(Intercept)	0.000	1	0.998
Leukoreduction moment	0.000	1	1.000
Blood component	0.000	1	1.000
Leukoreduction moment:blood component	0.000	1	1.000

X^2^ - Chi-Square; df - Degrees of Freedom;

* Generalized mixed-effects model for binomial family.

The data in Table 3 and Figure 1 show that the leukoreduction moment significantly influenced (p<0.009) the LOS, but this effect was not dependent on the transfused blood component (p=0.124). Patients who received pre-storage leukoreduced blood components had a shorter hospital stay (approximately 2 days) compared to the group that received post-storage leukoreduced blood components (p=0.010).

**Table 3:** Influence of the leukoreduction moment on the length of hospital stay, São Paulo, Brazil, 2017-2020

	X^2^	df	*p* value^*^
(Intercept)	75.447	1	< 0.001
Leukoreduction moment	6.729	1	0.009
Blood component	3.032	1	0.082
Leukoreduction moment:blood component	2.363	1	0.124

X^2^ - Chi-Square; df - Degrees of Freedom;

* Generalized mixed-effects model for binomial family.

For the analysis of in-hospital mortality, the model did not show an influence of the leukoreduction moment (p=0.769) or the blood component (p=0.984) on the outcome (Table 4 and Figure 1).

**Table 4:** Influence of the leukoreduction moment on the occurrence of in-hospital mortality, São Paulo, Brazil, 2017-2020

	X^2^	df	*p* value^*^
(Intercept)	42.074	1	<0.001
Leukoreduction moment	0.086	1	0.769
Blood component	0.019	1	0.892
Leukoreduction moment:blood component	0.000	1	0.984

X^2^ - Chi-Square; df - Degrees of Freedom;

* Generalized mixed-effects model for binomial family.

## DISCUSSION

This research included a specific cohort of patients who received leukoreduced RBCs or PCs in the emergency department, making this type of analysis unprecedented in both national and international literature. The study identified that the leukoreduction moment influenced the patients’ LOS and was independent of the transfused blood component; in other words, pre-storage leukoreduction performed better for both RBCs and PCs. For other outcomes analyzed (HAI, TR, and in-hospital mortality), no differences were found between patients who received allogeneic blood components based on the leukoreduction moment.

Although the literature shows positive evidence of the effect of leukoreduction moment on the occurrence of TRs^(14-15,17-19)^, this study did not find the same results, which could be associated with the characteristics of the sample (patients who received blood components in the emergency department). Moreover, the high number of TRs in the sample without a specific diagnosis and the absence of cases of circulatory overload indicate possible underreporting by professionals, a condition also reported in other hemovigilance studies^(21-22)^.

Regarding the outcome of HAI occurrence within 72 hours of transfusion, no association was found with the moment of filtration, similar to a Dutch study that analyzed infection cases in the postoperative period of cardiac surgery^(23)^. However, it is worth noting that the follow-up period considered by the researchers of this study was 60 days post-surgery^(23)^. Therefore, there is a need for further research to investigate the influence of leukoreduction moment on the occurrence of HAIs, considering time frames from at least 72 hours post-transfusion to longer periods, such as hospital discharge.

Contrary to the results found in the present study, researchers have shown that the LOS was similar between patients who received preand post-storage leukoreduced blood components^(23)^. For the mortality outcome, the results also showed no difference between the groups; however, the researchers did not clarify whether the death occurred during the hospital stay^(23)^, as considered in this study.

A more in-depth and comparative discussion of the results of this research with the scientific literature was not possible due to the scarcity of studies on the subject, the heterogeneity of research regarding patient inclusion criteria and analyzed outcomes, and the lack of studies exclusively investigating patients transfused in the emergency department. Another point to highlight is the limited number of recently published studies comparing the leukoreduction moment (the most recent reference identified in the literature dates back to 2018)^(14)^. This may be related to the implementation of universal leukoreduction (use of pre-storage filters for all patients) in the healthcare systems of European and North American countries, unlike in Brazil, where the indication for leukoreduction is still selective in institutions.

Brazilian legislation prioritizes certain groups for the indication of leukoreduced blood components, such as those who previously presented with FNHR or alloimmunization/HLA, bone marrow transplant recipients, patients with congenital immunodeficiency syndromes, and those with severe oncohematological diseases until the diagnosis is clarified^(24)^. This is directly related to the costly practice of leukoreduction for the Brazilian Unified Health System (SUS), especially pre-storage leukoreduction. However, it should be considered that the cost applied for the implementation and use of pre-storage leukoreduction (inline or bench) can be offset by the increased turnover rate of hospital beds, as evidenced by the shorter LOS of patients who received this type of filtration in the present study.

### Study limitations

The limitations of this study include the small sample size and the low frequency of events, which may have influenced the findings. Additionally, the study did not focus on investigating whether variables associated with previous conditions, such as the number of transfusions received during prior hospitalizations with or without the occurrence of TRs, have an impact on the clinical outcomes of patients transfused in the emergency department. Future studies should consider multicentric analyses involving institutions from different regions of Brazil, given the varying realities of the healthcare system across different cities and capitals in the country.

### Contributions to Nursing, Health, or Public Policy

Finally, this investigation’s results contribute to the clinical practice of the emergency team, particularly nurses^(25-26)^, who oversee bedside transfusions and can detect possible TRs and other associated complications early on. The findings also have implications for public hemotherapy policies concerning the leukoreduction procedure.

## CONCLUSIONS

The results of this study indicate that administering pre-storage leukoreduced RBCs and PCs to patients in the emergency department reduces hospital stay length. The occurrence of HAIs, TRs, and in-hospital mortality was not influenced by the leukoreduction moment, regardless of the type of blood component transfused. This suggests that other factors may affect the incidence of these outcomes, highlighting the importance of future investigations to improve clinical transfusion protocols.
