# Supplementary material for: Influence of the leukoreduction moment of blood components on the clinical outcomes of transfused patients in the emergency department
Source: Rev Bras Enferm. 2024 Aug 26;77(5):e20230293. doi: 10.1590/0034-7167-2023-0293 (PMC11346908; doi:10.1590/0034-7167-2023-0293)
Supplement: Translation

# Translation: Influência do momento da leucorredução de hemocomponentes na evolução clínica de pacientes transfundidos na emergência

**DOI:** 10.1590/0034-7167-2023-0293pt

**Authors:** Natasha Dejigov Monteiro da Silva, Ana Catharina Herbst, Milena Raquel André, Lilia de Souza Nogueira

**Affiliations:** I Universidade de São Paulo. São Paulo, São Paulo, Brasil

**Keywords:** Transfusão de Sangue, Procedimentos de Redução de Leucócitos, Reação Transfusional, Evolução Clínica, Serviços Médicos de Emergência

## Abstract

**Objetivos:**

verificar a influência do momento da leucorredução (pré ou pós-armazenamento) de hemocomponentes na evolução clínica de pacientes transfundidos na emergência.

**Métodos:**

coorte retrospectiva de pacientes com idade igual ou maior de 18 anos que receberam, no departamento de emergência, concentrado de hemácias ou plaquetas leucorreduzidas pré ou pós-armazenamento. Modelo de efeitos mistos generalizado foi aplicado nas análises.

**Resultados:**

na amostra de 373 pacientes (63,27% homens, idade média 54,83) e 643 transfusões (69,98% concentrado de hemácias) foi identificado que o momento da leucorredução influenciou o tempo de internação hospitalar dos pacientes (p<0,009), porém não foi dependente do hemocomponente transfundido (p=0,124). O momento da leucorredução não teve efeito (p>0,050) nas variáveis reação transfusional, infecção relacionada à assistência à saúde e óbito.

**Conclusões:**

pacientes que receberam na emergência hemocomponente leucorreduzido pré-armazenamento apresentaram menor tempo de internação hospitalar.

## INTRODUÇÃO

A transfusão de hemocomponentes é uma terapia relevante para o tratamento de pacientes, especialmente os críticos, com o objetivo de obter melhora clínica, como aumento na oxigenação tecidual, prevenção de hemorragia ou cessação do sangramento^(1-3)^. Os hemocomponentes são produtos gerados a partir do sangue total e classificados em concentrado de hemácias (CH), concentrado de plaquetas (CP), plasma fresco congelado (PFC) e crioprecipitado (Crio)^(2,4)^.

Durante as etapas de coleta, processamento e armazenamento do hemocomponente ocorre liberação de mediadores inflamatórios resultantes da degradação de leucócitos. No ato transfusional são introduzidos antígenos leucocitários e células metabolicamente ativas capazes de se proliferar e produzir modificadores imunológicos que afetam o receptor, que responderá à transfusão produzindo mediadores imunológicos próprios, com consequente ocorrência de reação transfusional (RT)^(4-7)^. Segundo a *Agence Régionale de Santé Île-de-France*, estima-se que a taxa de RTs seja de 3 a 5 por 1.000 transfusões realizadas^(8)^, sendo que os CH e CP são responsáveis pela maioria delas^(3,8-9)^.

A fim de reduzir efeitos adversos da transfusão, especialmente reação febril não hemolítica (RFNH), aloimunização ao sistema Antígeno Leucocitário Humano (ALO/HLA) e lesão pulmonar aguda relacionada à transfusão (*transfusion-related acute lung injury* - TRALI), um dos procedimentos adotados é a leucorredução de CH e CP que pode ser realizada por meio de filtros, em dois momentos: pré-armazenamento (bancada ou em linha) ou pós-armazenamento (beira leito)^(10-12)^. As filtrações de bancada e em linha são realizadas no Serviço de Hemoterapia em até 48 horas e entre 2 a 24 horas após a coleta, respectivamente. A filtração beira leito é realizada no momento do ato transfusional no paciente^(12)^. O PFC e o Crio não necessitam de leucorredução, pois não há quantidade suficiente de leucócitos na bolsa destes componentes que possa ser nocivo ao receptor.

Na emergência, o uso de hemocomponentes é frequente e se justifica pela necessidade de auxiliar na reposição volêmica, controlar o sangramento e restabelecer a oxigenação tecidual. Há evidências de benefícios da leucorredução realizada no momento pré-armazenamento em relação ao pós-armazenamento quanto à redução de ocorrência de RTs e infecção em pacientes transfundidos em diferentes setores do hospital^(13-19)^. Entretanto, análises de pacientes que receberam hemocomponentes leucorreduzidos no departamento de emergência são escassas na literatura.

Ressalta-se ainda que a atuação do enfermeiro e de toda a equipe assistencial durante a realização da transfusão de hemocomponentes é crucial para assegurar a segurança e eficácia do procedimento, especialmente de pacientes em situações críticas de saúde. Neste contexto, implantar processos que possibilitem a redução de ocorrência de efeitos adversos da transfusão não só trazem benefícios para o paciente, como também para o equipamento de saúde, com a redução da carga de trabalho da equipe assistencial, menor consumo de insumos para tratamento de ocorrências e, possivelmente, diminuição no tempo de internação hospitalar^(20)^.

Considerando a importância da leucorredução na redução de efeitos adversos da transfusão e a identificação de lacunas de conhecimento sobre o possível impacto do momento da leucorredução (pré ou pós-armazenamento) em desfechos clínicos de pacientes que receberam hemocomponente durante o atendimento de emergência, justifica-se a relevância da presente pesquisa.

## OBJETIVOS

Verificar a influência do momento da leucorredução de hemocomponentes na evolução clínica de pacientes transfundidos no departamento de emergência.

## MÉTODOS

### Aspectos éticos

A presente pesquisa foi aprovada pelos Comitês de Ética em Pesquisa das instituições participantes e, por se tratar de uma coleta retrospectiva com análise documental, houve dispensa da aplicação do Termo de Consentimento Livre e Esclarecido, com a garantia de que os pesquisadores mantivessem o sigilo e a confiabilidade das informações coletadas.

### Desenho, período e local do estudo

Trata-se de um estudo de coorte retrospectivo, norteado pela ferramenta *STrengthening the Reporting of OBservational studies in Epidemiology* (STROBE). A coleta de dados foi realizada em três Institutos do Hospital das Clínicas da Faculdade de Medicina da Universidade de São Paulo (HCFMUSP): Instituto do Câncer do Estado de São Paulo (ICESP), especializado em oncologia; Instituto do Coração (InCor), especializado em cardiopneumologia; e, Instituto Central (ICHC), que atende várias especialidades e é centro de referência para atendimento ao traumatizado.

### População ou amostra; critérios de inclusão e exclusão

A amostra, por conveniência, incluiu pacientes com idade igual ou superior a 18 anos que receberam, no departamento de emergência de um dos Institutos, transfusão de CH e/ou CP alogênico leucorreduzido pré ou pós-armazenamento, no período de 01 de janeiro de 2017 a 31 de dezembro de 2020, e que permaneceram por, no mínimo, 24 horas internados. Pacientes com ordem de não reanimar, em morte encefálica e/ou com diagnóstico de sepse na admissão hospitalar foram excluídos da amostra.

### Protocolo do estudo

A variável independente do estudo foi o momento da leucorredução, categorizado em pré-armazenamento (em linha ou bancada) ou pós-armazenamento (beira leito). Para as variáveis dependentes relacionadas à evolução clínica dos pacientes foram consideradas a ocorrência de RTs (categorizada em sim ou não a partir da identificação de RFNH, hipotensão relacionada à transfusão, TRALI, ALO/HLA ou sintomas relacionados à transfusão); presença de infecção relacionada à assistência à saúde (IRAS) em até 72 horas após a transfusão (categorizada em sim ou não); tempo de internação hospitalar (considerado em dias) e óbito hospitalar (categorizado em sim ou não).

Para a operacionalização da coleta de dados, primeiramente foi solicitada ao Banco de Sangue da Fundação Pró-Sangue do Hemocentro de São Paulo (FPS-HSP) o banco de dados das requisições e dispensas de hemocomponentes realizadas no período de interesse da presente pesquisa, de onde foram extraídas as informações referentes aos hemocomponentes e presença de transfusões e reações prévias dos pacientes. Esta base foi cruzada com o banco de dados dos prontuários eletrônicos dos pacientes admitidos nos institutos participantes, os critérios de elegibilidade do estudo foram aplicados e as variáveis de interesse da pesquisa coletadas.

Cabe ressaltar que a filtração pré-armazenamento (em linha ou bancada) foi realizada na FPS-HSP. Para a filtração em linha, foi utilizado o sistema de coleta automática de sangue Trima Accel^®^ com capacidade de filtração maior que 5 x 10^6 de leucócitos por unidade. Na filtração de bancada, foi utilizado o filtro BIOR 01 PLUS BBS PF (para CH) e BIOP 10 Plus BBSS PF (para CP), ambos da Fresenius^®^ e com nível residual de leucócitos inferior a 2 x 10^5 por unidade. A filtração beira leito foi realizada nos Institutos, no momento do ato transfusional, por meio do filtro RC1VAE (Auto Prime) da Haemonetics^®^ (para CH), com capacidade residual menor de 2 x 10^5 de leucócitos por unidade, e do filtro PL3VAE da Haemonetics^®^ (para CP) com recuperação de plaquetas superior a 90% e nível de leucócitos residuais inferior a 2 x 10^5 por transfusão.

A busca por informações sobre a ocorrência de RTs foi realizada no Sistema de Notificação em Vigilância Sanitária (NOTIVISA) e no Centro de Vigilância Sanitária (CVS-4), que contêm o registro obrigatório do destino final dos hemocomponentes preparados para a transfusão, além dos prontuários eletrônicos dos pacientes. As informações sobre IRAS foram obtidas por meio da busca de registro de exames de cultura realizados em até 72 horas após a transfusão e a validação dos critérios de infecção foi realizada junto às Comissões de Controle de Infecção Hospitalar dos Institutos. O tempo de internação e a condição de saída hospitalar foram identificados nos prontuários eletrônicos dos pacientes.

Considerando a complexidade do resgate das informações, que envolveu a consulta a diferentes fontes, o período de abrangência (2017 a 2020) e as restrições de acesso aos Institutos devido à pandemia da COVID-19, a coleta dos dados teve duração de dois anos (2021 a 2022).

### Análise dos resultados e estatística

Os dados coletados foram inseridos no programa *Microsoft Excel* ^®^ 2019 e analisados por um profissional estatístico no programa R versão 4.3.1. Considerando que cada paciente pode ter recebido mais de uma transfusão durante a permanência no departamento de emergência, o modelo de efeitos mistos generalizado para família binomial foi aplicado para verificar a influência do momento da leucorredução para cada variável dependente do estudo, controlado pelo hemocomponente transfundido (CH ou CP). O nível de significância adotado em todas as análises foi de 5%.

## RESULTADOS

No período do estudo, 373 pacientes receberam hemocomponentes no departamento de emergência, sendo a maioria do sexo masculino (63,27%), da cor branca (68,84%) e com idade média e mediana de 54,83 e 58 anos, respectivamente. Em relação ao diagnóstico de admissão na emergência, prevaleceram as neoplasias (n=118; 31,63%) e as doenças do sangue e dos órgãos hematopoiéticos (n=112; 30,03%). O tipo sanguíneo mais incidente foi O fator Rh positivo ou negativo (46,92%) seguido do A fator Rh positivo ou negativo (37,26%). Um total de 303 (81,23%) pacientes já havia recebido transfusão previamente e, destes, 269 (88,78%) relataram já ter apresentado alguma RT.

Das 643 transfusões realizadas no departamento de emergência, o hemocomponente mais administrado foi o CH (69,98%) e o filtro beira leito o mais utilizado (86,47%). Das 144 justificativas transfusionais descritas, foram mais prevalentes a anemia (61,80%) seguida de sangramento ativo (27,08%). A média de transfusões por paciente foi de 1,45 (DP 0,93).

Foram identificadas na amostra 52 (8,09%) RTs, sendo 41 descritas como RT sem diagnóstico definido, 8 reações alérgicas, 2 RFNH e 1 dispneia associada à transfusão. Um total de 6 pacientes apresentou IRAS em até 72 horas da transfusão, com destaque para a infecção do trato urinário. O tempo médio de internação hospitalar foi de 4,98 (DP 8,18) dias, com variação de 0,57 a 88,47 dias. Em relação ao desfecho hospitalar, 35 (9,38%) pacientes não sobreviveram.

Na aplicação do modelo de efeitos mistos generalizado, foi considerado o conjunto (cluster) de 373 pacientes para as 643 observações de transfusões realizadas. Os dados das Tabelas 1 e Figura 1 mostram que não há evidência significativa de que o momento da leucorredução tenha efeito na ocorrência de RT (p=0,786), independente do hemocomponente transfundido (p=0,796).

**Tabela 1:** Influência do momento da leucorredução na ocorrência de reação transfusional, São Paulo, Brasil, 2017-2020

	X^2^	gl	Valor de *p* ^*^
(Intercepto)	41,152	1	<0,001
Momento da leucorredução	0,074	1	0,786
Hemocomponente	1,263	1	0,261
Momento da leucorredução:hemocomponente	0,067	1	0,796

X^2^ - Qui-Quadrado; gl- grau de liberdade;

* Modelo de efeitos mistos generalizado para família binomial.

*RT - Reação transfusional; IRAS - Infecção relacionada à assistência à saúde.*

**Figura 1:** Estimativas de reação transfusional, infecção relacionada à assistência à saúde, tempo de internação e óbito hospitalar segundo momento da leucorredução e tipo de hemocomponente, São Paulo, Brasil, 2017-2020 RT – Reação transfusional; IRAS – Infecção relacionada à assistência à saúde.

Para a análise de presença de IRAS em até 72 horas após a transfusão, os achados evidenciaram que o efeito do momento da leucorredução para o desfecho foi semelhante (p=1,000), independente do hemocomponente administrado (p=1,000) (Tabela 2, Figura 1).

**Tabela 2:** Influência do momento da leucorredução na presença de infecção relacionada à assistência à saúde, São Paulo, Brasil, 2017-2020

	X^2^	gl	Valor de *p* ^*^
(Intercepto)	0,000	1	0,998
Momento da leucorredução	0,000	1	1,000
Hemocomponente	0,000	1	1,000
Momento da leucorredução:hemocomponente	0,000	1	1,000

X^2^ - Qui-Quadrado; gl- grau de liberdade;

* Modelo de efeitos mistos generalizado para família binomial.

Os dados da Tabela 3 e Figura 1 evidenciam que o momento da leucorredução exerceu influência (p<0,009) no tempo de internação hospitalar dos pacientes, porém não foi dependente do hemocomponente transfundido (p=0,124). Os pacientes que receberam hemocomponente leucorreduzido pré-armazenamento apresentaram menor tempo de internação hospitalar (aproximadamente 2 dias) quando comparado ao grupo que recebeu hemocomponente leucorreduzido pós-armazenamento (p=0,010).

**Tabela 3:** Influência do momento da leucorredução no tempo de internação hospitalar, São Paulo, Brasil, 2017-2020

	X^2^	gl	Valor de *p* ^*^
(Intercepto)	75,447	1	< 0,001
Momento da leucorredução	6,729	1	0,009
Hemocomponente	3,032	1	0,082
Momento da leucorredução:hemocomponente	2,363	1	0,124

X^2^ - Qui-Quadrado; gl- grau de liberdade;

* Modelo de efeitos mistos generalizado para família binomial.

Para a análise de óbito hospitalar, o modelo não evidenciou influência do momento da leucorredução (p=0,769) e nem do hemocomponente (p=0,984) sobre o desfecho (Tabela 4 e Figura 1).

**Tabela 4:** Influência do momento da leucorredução na ocorrência de óbito hospitalar, São Paulo, Brasil, 2017-2020

	X^2^	gl	Valor de *p* ^*^
(Intercepto)	42,074	1	<0,001
Momento da leucorredução	0,086	1	0,769
Hemocomponente	0,019	1	0,892
Momento da leucorredução:hemocomponente	0,000	1	0,984

X^2^ - Qui-Quadrado; gl- grau de liberdade;

* Modelo de efeitos mistos generalizado para família binomial.

## DISCUSSÃO

A presente pesquisa incluiu uma coorte específica de pacientes que receberam, no departamento de emergência, CH ou CP leucorreduzidos, sendo este tipo de análise inédito na literatura nacional e internacional. Na amostra, foi identificado que o momento da leucorredução influenciou o tempo de internação hospitalar dos pacientes e foi independente do hemocomponente transfundido, ou seja, a leucorredução pré-armazenamento teve melhor desempenho tanto para CH quanto para CP. Quanto aos outros desfechos analisados (IRAS, RT e óbito hospitalar), não foi identificada diferença entre os pacientes que receberam hemocomponentes alogênicos segundo o momento da leucorredução.

Apesar de a literatura mostrar evidências positivas do efeito do momento da leucorredução na ocorrência de RT^(14-15,17-19)^, este estudo não encontrou os mesmos resultados, o que pode estar associado às características da amostra (pacientes que receberam hemocomponentes no departamento de emergência). Ademais, o elevado número de RTs na amostra sem descrição de diagnóstico e a ausência de casos de sobrecarga circulatória demonstram possível subnotificação dos profissionais, condição também relatada em outros estudos sobre hemovigilância^(21-22)^.

Com relação ao desfecho presença de IRAS em até 72 horas da transfusão, não foi encontrada associação com o momento de filtração, semelhante ao estudo holandês que analisou casos de infecção no pós-operatório de cirurgia cardíaca^(23)^. Entretanto, vale destacar que o tempo de seguimento considerado pelos pesquisadores deste estudo foi de 60 dias após a cirurgia^(23)^. Observa-se, assim, a necessidade de outras pesquisas que investiguem a influência do momento da leucorredução na ocorrência de IRAS considerando recortes temporais de, no mínimo, 72 horas após a transfusão até períodos mais longos, como o momento da saída hospitalar.

Diferentemente do resultado encontrado no presente estudo, pesquisadores evidenciaram que o tempo de internação hospitalar foi semelhante entre os pacientes que receberam hemocomponentes leucorreduzidos pré e pós-armazenamento^(23)^. Para o desfecho óbito, os resultados encontrados também não mostraram diferença entre os grupos; entretanto, os pesquisadores não deixam claro se a morte ocorreu durante a internação hospitalar^(23)^, como considerada nesta pesquisa.

Uma discussão mais aprofundada e comparativa dos resultados da presente pesquisa com a literatura científica não foi possível de ser realizada frente à escassez de pesquisas sobre o tema, heterogeneidade das pesquisas quanto aos critérios de inclusão dos pacientes e desfechos analisados, além da ausência de estudos que investigaram exclusivamente pacientes transfundidos no departamento de emergência. Outro ponto a ser destacado é o reduzido número de pesquisas recentemente publicadas que compararam o momento da leucorredução (a referência mais nova identificada na literatura data de 2018)^(14)^. Tal fato pode estar relacionado à implementação da leucorredução universal (uso de filtros de pré-armazenagem para todos os pacientes) nos sistemas de saúde de países da Europa e América do Norte, diferente do Brasil, onde a indicação da leucorredução ainda é seletiva nas instituições.

A legislação brasileira orienta a priorização de alguns grupos na indicação de hemocomponentes leucorreduzidos, como, por exemplo, aqueles que apresentaram previamente RFNH ou ALO/HLA, transplantados de medula óssea, com síndromes de imunodeficiências congênitas e doenças oncohematológicas graves até o esclarecimento de diagnóstico^(24)^. Tal fato está diretamente relacionado à prática custosa da leucorredução para o Sistema Único de Saúde, especialmente a do tipo pré-armazenamento. Entretanto, deve-se considerar que o custo aplicado para a implantação e uso da leucorredução pré-armazenamento (em linha ou bancada) pode ser compensado pelo aumento do índice de rotatividade de leitos, conforme evidenciado pelo menor tempo de internação dos pacientes que utilizaram este tipo de filtração no presente estudo.

### Limitações do estudo

Como limitações deste estudo, destacam-se o reduzido tamanho da amostra e a baixa frequência de eventos que podem ter influenciado os achados. Além disso, não foi foco do estudo investigar se variáveis associadas às condições prévias, como número de transfusões recebidas em internações anteriores com ou sem ocorrência de RTs, têm repercussão na evolução clínica dos pacientes transfundidos na emergência. Sugere-se, como propostas de estudos futuros, a realização de análises multicêntricas que envolvam instituições de diferentes regiões do Brasil, visto que a realidade do sistema de saúde difere entre cidades e capitais do país.

### Contribuições para a área da Enfermagem, Saúde ou Política Pública

Por fim, destaca-se a contribuição dos resultados desta investigação para a prática clínica da equipe da emergência, especialmente do enfermeiro^(25-26)^ que acompanha à beira do leito a transfusão nos pacientes e detecta precocemente possíveis RTs e outras complicações associadas, e com políticas públicas de hemoterapia no que tange ao procedimento de leucorredução.

## CONCLUSÕES

Os resultados encontrados neste estudo permitiram concluir que a administração de CH e CP leucorreduzidos pré-armazenamento em pacientes no departamento de emergência reduz o tempo de internação hospitalar. A ocorrência de IRAS, RT e óbito hospitalar não foi influenciada pelo momento da leucorredução, independente do tipo de hemocomponente transfundido. Isto sugere que outros fatores podem influenciar a incidência destes desfechos, evidenciando a importância de futuras investigações para o aprimoramento de protocolos clínicos transfusionais.
